# Strategic supplementation of *Flemingia* silage to enhance rumen fermentation efficiency, microbial protein synthesis and methane mitigation in beef cattle

**DOI:** 10.1186/s12917-020-02703-x

**Published:** 2020-12-09

**Authors:** Bounnaxay Viennasay, Metha Wanapat

**Affiliations:** Tropical Feed Resources Research and Development Centre (TROFREC), Department of Animal Science, Faculty of Agriculture, Khon Kaen University, Khon Kaen, 40002 Thailand

**Keywords:** Fodder silage, Feed utilization, Rumen metabolism, Methane production, Phytonutrients

## Abstract

**Background:**

Good quality protein as an on-farm feed resource has been in great demand to support the productivity of ruminants. A digestion trial using beef cattle crossbreds was conducted to assess the four dietary treatments of *Flemingia macrophylla* silage (FMS) supplementation at 0, 0.2, 0.4 and 0.6 kg dry matter (DM)/day in a 4 × 4 Latin square design. Feed DM intakes were measured during the 14 days and sample of feeds, feces, urine, as well as rumen fluid and blood were collected during the 7 days while the animals were on metabolism crates.

**Results:**

Based on this experiment strategic supplementation of FMS increased (*P* < 0.05) nutrients digestibility (organic matter, crude protein, and acid detergent fiber) enhanced rumen total volatile fatty acid production especially propionic acid (C_3_), C_2_:C_3_ ratio while, remarkably promoted the microbial protein synthesis (MPS) by increasing N-balance and retention of purine derivatives.

**Conclusions:**

Under this experiment, the results revealed the potential use of FMS as a good-quality feed to improve nutrients digestibility, rumen fermentation, microbial protein synthesis, and to mitigate methane production. FMS supplementation at 0.6 kg DM/day exhibited the best result.

## Background

Feed resources for ruminants are important in the livestock feeding systems for small-scale tropical farmers; particularly in the dry season [1]. *Flemingia* is a multipurpose legume shrub that yields fresh biomass of about 55 tons/ha/year and thrives well in diverse conditions [2]. It contains high levels of crude protein (17–26%), condensed tannins (CT) (6–11%) and saponins (SPN) [3–5]. The presence of these phytonutrients in feed resources has been shown to enhance the rumen fermentation efficiency and greatly reduce rumen methane (CH_4_) production [6]. Fagundes et al. [7] also reported that supplementation of *Flemingia* at 125 g of dry matter intake in goats did not affect adversely the feed intake and milk production. In addition [8], it was reported that supplemented *Flemingia* hay meal at 150 g/head/day increased digestibility of nutrients, rumen fermentation and microbial protein synthesis. Moreover, Kang et al. [4] confirmed that *Flemingia* leaves supplementation improved rumen fermentation and reduced the CH_4_ production. Conservation of feed in the form of silage has been a good practice especially for dry season feeding [9]. Silage quality can be enhanced by addition of urea and molasses in fodder crop silage [10–12].

However, there is limited information about the utilization of *Flemingia macrophylla* silage on rumen fermentation. Hence, the aim of this experiment was to investigate the impact of *Flemingia macrophylla* silage on nutrient digestibility, rumen fermentation and microbial protein synthesis in beef cattle.

## Results

### Nutritive value, feed intake and nutrient digestibility

Concentrate supplement was formulated using cassava chip and agricultural by-products, namely rice bran, palm kernel meal, molasses etc., as shown in Table 1. The nutritive values of the feeds were in good ranges especially the FMS which had a good characteristic of silage both physically and chemically.

**Table 1:** Feed ingredients and chemical composition of experimental diets

Items	Concentrate	Rice straw	*Flemingia macrophylla* silage (FMS)
Ingredients (% air-dry basis)			
Cassava chip	60.00	0.00	0.00
Brewery’s grain, dried	12.00	0.00	0.00
Rice bran	9.00	0.00	0.00
Palm kernel meal	13.00	0.00	0.00
Urea	2.00	0.00	0.00
Molasses	2.00	0.00	0.00
Sulfur	0.50	0.00	0.00
Salt	1.00	0.00	0.00
Mineral premix	0.50	0.00	0.00
Chemical composition (% DM)			
Dry matter	87.50	90.00	26.50
Organic matter	14.80	2.80	18.10
Crude protein	94.20	96.40	95.60
Neutral detergent fiber	28.90	71.70	47.50
Acid detergent fiber	17.20	47.70	37.20
Condensed tannins	0.00	0.00	10.20
pH	0.00	0.00	4.40
Lactic acid (g/L)	0.00	0.00	2.00
Acetic acid (g/L)	0.00	0.00	0.50

Strategic supplementation of FMS did not influence the total feed intake (*P* > 0.05), however nutrients digestibilities of DM, OM, CP, NDF, and ADF were significantly increased (*P* < 0.001) (Table 2). In addition, the supplementation of FMS at 0.6 kg/head/day resulted in the highest in nutrients digestibilities.

**Table 2:** Effect of *Flemingia macrophylla* silage (FMS) on feed intake and nutrients digestibility

Items	FMS kg/d of dry mater				SEM	*P-*value
	0	0.2	0.4	0.6		
Roughage Intake (DM basis)						
Kg/d	3.13 ± 0.84	3.23 ± 0.97	3.40 ± 0.89	3.58 ± 0.70	1.00	0.47
Body weight (%)	1.81 ± 0.25	1.97 ± 0.22	1.89 ± 0.34	2.14 ± 0.11	0.60	0.44
Concentrate Intake						
kg/d	0.90 ± 0.17	0.90 ± 0.17	0.90 ± 0.17	0.90 ± 0.17	0.00	0.00
Body weight (%)	0.50 ± 0.03	0.50 ± 0.03	0.50 ± 0.03	0.50 ± 0.03	0.00	0.00
Total Intake						
Kg/d	4.00 ± 0.98	4.30 ± 1.13	4.10 ± 1.03	4.50 ± 0.85	1.00	0.48
Body weight (%)	2.32 ± 0.24	2.48 ± 0.22	2.41 ± 0.35	2.60 ± 0.21	0.06	0.44
Digestibility (%)						
Dry matter	54.11 ± 1.49^a^	58.61 ± 0.68^b^	62.30 ± 2.57^c^	63.62 ± 2.49^c^	0.21	0.001
Organic matter	58.20 ± 0.46^a^	64.40 ± 0.42^b^	68.40 ± 2.10^c^	71.45 ± 0.66^d^	0.29	0.001
Crude protein	53.80 ± 0.65^a^	55.60 ± 0.32^b^	57.10 ± 0.17^c^	58.70 ± 0.32^d^	0.10	0.001
Neutral detergent fiber	50.00 ± 0.81^a^	51.90 ± 0.92^ab^	54.00 ± 0.56^ab^	55.40 ± 1.89^c^	0.12	0.05
Acid detergent fiber	45.80 ± 0.28^a^	47.30 ± 0.54^b^	49.60 ± 0.88^c^	52.80 ± 0.54^d^	0.15	0.001

^a,b,c,d^Means in the same row with different superscripts differ (*P* < 0.01), *SEM* Standard error of the mean

### Rumen fermentation efficiency and blood urea nitrogen

FMS supplementation increased total VFA and propionic acid (C_3_) (*P* < 0.001), among treatments (Table 3). While the concentrations of NH_3_–N, acetic acid (C_3_), butyric acid (C_4_), acetic acid to propionic acid (C_2_ to C_3_), CH_4_ production, and protozoal population were decreased (*P* < 0.001) among treatments, respectively. However, ruminal pH and BUN were not changed.

**Table 3:** Effect of *Flemingia macrophylla* silage (FMS) on rumen ecology and fermentation

Items	FMS kg/d of dry mater				SEM	*P-*value
	0	0.2	0.4	0.6		
NH_3_-N (mg/ml)	19.31 ± 0.12^d^	18.32 ± 0.13^c^	17.53 ± 0.11^b^	16.91 ± 0.08^a^	0.50	0.001
BUN (mg/dl)	10.11 ± 0.13	10.03 ± 0.21	10.21 ± 0.43	10.03 ± 0.38	0.02	0.83
Total VFA (mM/L)	99.50 ± 4.61^a^	102.90 ± 4.61^ab^	107.70 ± 3.52^bc^	112.50 ± 4.23^c^	2.82	0.004
VFA (mol/100 mol)						
Acetic acid	73.90 ± 0.67^d^	73.00 ± 0.36^c^	71.70 ± 0.94^b^	70.20 ± 0.98^a^	0.81	0.001
Propionic acid	16.50 ± 0.20^a^	17.70 ± 0.19^b^	19.30 ± 0.59^c^	20.90 ± 0.74^d^	0.95	0.001
Butyric acid	9.70 ± 0.53^c^	9.20 ± 0.33^b^	9.00 ± 0.39^ab^	8.90 ± 0.26^a^	0.18	0.002
Acetic acid to Propionic acid	4.50 ± 0.08^d^	4.10 ± 0.06^c^	3.80 ± 0.16^b^	3.40 ± 0.16^a^	0.23	0.001
Methane (mol/100 mol)	32.60 ± 0.16^d^	31.68 ± 0.14^c^	30.59 ± 0.45^b^	29.38 ± 0.55^a^	0.69	0.001
pH	6.82 ± 0.03	6.87 ± 0.02	6.84 ± 0.03	6.89 ± 0.02	0.02	0.13
Protozoa (×10^5^Cell/ml)	8.08 ± 0.07^c^	6.65 ± 0.05^b^	4.98 ± 0.07^a^	4.40 ± 0.09^a^	0.83	0.009

^a,b,c,d^Means in the same row with different superscripts differ (*P* < 0.01), *SEM* Standard error of the mean, *BUN* Blood urea nitrogen, *VFA* Volatile fatty acid, Methane production=0.45 (acetic acid) – 0.275 (propionic acid) + 0.4 (butyric acid)

### Nitrogen balance, excretion of purine derivatives and microbial nitrogen supply

Table 4, shows nitrogen intake ranged from 34.81 ± 1.23 to 48.01 ± 2.12 g/d and was increased (*P* < 0.001), while N excretion was similar among treatments (*P* > 0.05). However, N absorbed and N retained were increased (*P* < 0.001), respectively. The FMS supplementation affected on allantoin, uric acid, PD, purine absorb, microbial nitrogen supply and EMNS (*P* < 0.001). Moreover, FMS affected on percent of MNS and EMNS were increased significantly among treatments (*P* < 0.001), respectively.

**Table 4:** Effects of *Flemingia macrophylla* silage (FMS) on nitrogen balance, excretion of purine derivatives and microbial nitrogen supply

Items	FMS kg/d of dry mater				SEM	*P-*value
	0	0.2	0.4	0.6		
Nitrogen utilization (g/d)						
Intake	34.81 ± 1.23^a^	41.02 ± 1.54^b^	44.03 ± 1.98^c^	48.01 ± 2.12^d^	2.79	0.001
N excretion (g/d)						
Fecal N	15.20 ± 2.32	16.00 ± 1.98	16.20 ± 1.67	13.90 ± 1.59	0.52	0.54
Urinal N	13.00 ± 1.41	13.40 ± 1.50	11.80 ± 1.83	10.50 ± 1.92	0.65	0.25
N balance (g/d)						
Absorbed N	19.61 ± 1.04^a^	25.02 ± 1.67^b^	28.01 ± 1.91^b^	34.10 ± 1.98^c^	3.02	0.001
Retained N	6.70 ± 0.54^a^	11.61 ± 0.946^b^	17.63 ± 1.32^c^	22.30 ± 1.27^d^	3.41	0.001
Allantoin (mM/d)	108.92 ± 3.54^a^	122.61 ± 4.21^b^	130.67 ± 5.33^b^	148.71 ± 8.12^c^	8.30	0.001
Uric acid (mM/d)	26.11 ± 1.34^a^	29.42 ± 2.30^b^	31.33 ± 1.11^b^	35.56 ± 1.72^c^	1.99	0.001
PD (mM/d)	130.67 ± 3.43^a^	147.22 ± 2.31^b^	156.78 ± 3.16^b^	178.43 ± 2.81^c^	9.96	0.001
Purine absorb (mM/d)	109.3 ± 2.43^a^	125.7 ± 2.56^b^	135.4 ± 2.11^b^	157.0 ± 2.43^c^	9.96	0.001
MNS (g N/d)	79.41 ± 2.21^a^	91.4 ± 3.11^b^	98.4 ± 4.01^b^	114.1 ± 4.87^c^	7.24	0.001
MNS increased (%)	0.00 ± 0.00^a^	15.43 ± 1.65^b^	24.12 ± 1.93^b^	44.01 ± 2.88^c^	9.20	0.002
EMNS (g N/kg OMDR)	9.60 ± 0.13^a^	11.31 ± 0.46^b^	14.13 ± 0.72^c^	14.12 ± 0.54^c^	1.01	0.003
EMNS increased (%)	0.00 ± 0.00^a^	18.92 ± 1.63^b^	39.78 ± 1.97^b^	49.01 ± 2.41^c^	10.9	0.001

^a,b,c^ Means in the same row with different superscripts differ (*P* < 0.05), *SEM* Standard error of the mean, *N* Nitrogen, *MNS* Microbial nitrogen supply, *PD* Purine derivatives. Microbial N (g N/d) = (X×70)/(0.116×0.83×1,000) = 0.727×X (where, X = total absorption of purine derivatives). EMNS = Efficiency of microbial nitrogen supply (g N/kg OMDR). OMDR (kg) = 65% of organic matter digestible in total tract

## Discussion

### Chemical composition of feed dry matter intake and digestibility of nutrients

As shown in Table 1, the nutritive value of rice straw obtained under this experiment had a low CP content and a high level of cell wall. These findings were similar to the values by Wanapat et al. [13]. Details of rice straw and the enhancement of nutritive value by various treatments had been illustrated by Wanapat et al. [14]. *Flemingia* is the shrub which can produce biomass for ruminant feeding. It contains high level of crude protein, CT and SP. The feed can be ensiled as silage for long time feeding, details are shown in Table 1, FMS contains 18.10% CP, 95.60% OM, 47.50% NDF, 37.20% ADF, and 10.20% CT, with pH of 4.4 and good characteristics. As reported by [15–17] who indicated that the pH of good silage should be 3.5 to 4.5. These results have shown that FMS was a good alternative feed to improve the quality and for a long dry season feeding. Providing additional source of energy such as molasses and urea as a non-protein nitrogen will lead to a higher CP content of the silage [18]. Feeding this silage with higher CP content would enrich the overall utilization especially when fed with low-quality roughages. Under this study, there were no differences in DM intakes among treatments (*P* < 0.05), but enhanced the digestibilities of OM, CP, NDF and ADF. Higher level of CP of the silage could have attributed the degradation activity of the rumen microbiomes. Phesatcha et al. [8] found similar results. It has been reported that CT in the feeds combined with protein to protect protein degradability in the rumen [19]. Tannin-protein feed complex would be available more in the lower-gut.

### Rumen Volatile Fatty Acids (VFA) and Blood-Urea-Nitrogen (BUN)

Ruminal pH and BUN were not significantly shifted differently among treatments. This result was similar to the findings of Phesatcha et al. [8] who reported *Flemingia* leaf supplementation did not change ruminal pH and BUN. The normal rumen pH was reported to be 6.3–6.8 which can support cellulolytic bacteria’s normal activity [20]. The tannin-protein complex in rumen could result in lower rumen NH_3_-N and enhance the protein availability in lower-gut [21, 22]. Ruminal NH_3_-N concentration was a key factor (15–30 mg/ml) for efficient microbial protein synthesis [23]. In this study, the ruminal NH_3_-N values (16.91 ± 0.08 to 19.31 ± 0.12 mg/ml) were found and could improve for rumen ecology in cattle crossbreds. With increased levels of FMS supplementation, the total VFA and C_3_ were remarkably increased (*P <* 0.05), and the highest impact was found in the group fed with of 0.6 kg/day, while C_2_, C_2_ to C_3_ ratio were reduced. Under the work of Phesatcha et al. [8], it was revealed that the ruminal C_2_ was reduced, which C_3_ was increased (*P <* 0.05). Makkar et al. [24] further showed that feeds containing condensed tannins (CT) could lower the ruminal C_2_ concentration. It was additionally reported that CT can impact on methanogenesis by reducing protozoa and methanogens, when C_2_ was reduced but C_3_ was greatly enhanced [25]. Another possible influence of rumen CH_4_ depression could be influenced by the suppression of protozoal population by FMS supplementation. This may be attributed to CT in FMS which could interfere the cell membrane of protozoa, thus interfering with ion exchanges [26]. Poungchompu et al. [27] earlier reported that dairy heifer crossbreds supplemented with feeds containing phytonutrients had reduced protozoal count. The population of rumen protozoal and methane emission were significantly reduced [28]. Earlier reports revealed that supplementation of plant secondary compounds, especially condensed tannins and saponins, could remarkably reduce protozoal population and methanogens in ruminants. However, the effect depended on dose-response of 1–2% of dry matter intake. Possible modes of action could be due to the direct effect of tannins on physical coating of protozoa whilst, saponins formed the sterol-biding with cell membrane of protozoa causing the destruction and blockage of ion-exchanges. Hence, such phenomenon caused the lysis of protozoa and methanogens [29–31]. However, in another study, it was shown that PTN could increase the population of *Fibrobacter succinogenes*, while the other two fibrolytic bacteria; *Ruminococcus albus and Ruminococcus flavefaciens* were decreased, but the actual mode of action needs to be further elucidated [32].

### Nitrogen balance, excretion of purine derivatives and microbial protein synthesis

FMS supplementation affected N-balance as shown in Table 4. The N-absorbed and retention were linearly increased with FMS supplementation due to N-intake and CP digestibility, however N-excretion of fecal and urine were similar among treatments. Agreed with, Viennasay et al. [12] who showed that the N-balance was improve when the digestibility of CP was high. In addition, rumen tannin-protein complex can support more protein available in lower-gut [33]. As shown, microbial protein synthesis in the rumen was a good indicator for efficient protein synthesis to enhance the overall protein utilization by the host ruminants [34]. Ruminal NH_3_-N concentration has been shown to support the microbial protein synthesis, as it was well-utilized by cellulolytic bacteria as an important source of nitrogen [35]. In adequate doses, the efficiency in microbial synthesis and the microbial yield were increased by including saponins [36] or CT [37] in the diets. Under this study, supplementation of FMS containing both CT and SPN, could provide additional protein available at the lower-gut for the host ruminants, as well as enhancing rumen fermentation efficiency.

## Conclusions

Under this experiment, the results revealed the potential use of *Flemingia macrophylla* silage as a good-quality feed to improve nutrients digestibility, rumen fermentation, microbial protein synthesis, and to mitigate methane production. *Flemingia macrophylla* silage supplementation at 0.6 kg DM/day exhibited the best result. Making *Flemingia macrophylla* silage should be encouraged to prepare for on-farm use especially during the long dry season. Furthermore, *in vivo* feeding trials should be conducted in both beef cattle and dairy cattle in order to obtain more relevant data.

## Methods

This experiment approval was granted by the Institutional Animal Care and Use Committee of Khon Kaen University, Thailand (Record no. IACUC-KKU-94/61 and reference no. 0201.2. 11/73).

### Preparation of *Flemingia macrophylla* silage

*Flemingia macrophylla* was planted by stems on the experimental plots of Tropical Feed Resources Research and Development Center (TROFREC), Department of Animal Science, Faculty of Agriculture, Khon Kaen University, Thailand with close supervision of the advisory Professor. All plant parts were kept and stored at TROFREC. *Flemingia macrophylla* (FM) whole top plant was harvested from the shrub after three months of regrowth. Silage of *Flemingia* was prepared by using young-whole leaf and stem, chopped (3 cm) and mixed with solution. Chopped fresh *Flemingia* (100 kg) was well-mixed with solution containing molasses, urea and water at 2:1:10, respectively. The mixture was ensiled in a plastic barrel for 21 days before feeding. Samples of FMS was randomly collected and later was analyzed for chemical compositions [38, 39], condensed tannins by methods [40, 41]. Apart from that, a FMS sample was washed with deionizing water for analyzed lactic acid and acetic acid analysis [42].

### Sample size

The sample size calculation was based on experimental design according to in a 4 × 4 Latin square design with 4 replicates, which provided a total of 16 experimental units.

### Inclusion and exclusion criteria

Neither inclusion nor exclusion was used, since the four beef cattle were similar in age, weight and pre-fed under similar feeding condition.

### Blinding

No blinding was performed, as the four treatments were already randomized statistically.

### Animals and design

These experimental beef crossbreds belonged to Tropical Feed Resource Research and Development center (TROFREC), Khon Kaen University and were provided as experimental animals for Ph.D. students. The animals were well-maintained for their health with good feeding and other management. After the experiment all animals were kept and maintained well with all aspects; health, nutrition, feeding and would be used later for other experiments. Four, beef cattle about two year old with 172 ± 43 kg liveweight, were randomly assigned to in a 4 × 4 Latin square design. Concentrate was offered at 0.5 kg of body weight (BW)/day and rice straw offered *ad libitum* with supplementation of FMS at 0, 0.2, 0.4 and 0.6 kg DM/head/day. The trial was conducted for four periods each was consisted of 21 days, during the first 14 days was for matter feed intake measurement, while during the last 7 days for sample collection using total collection method. Each animal was in individual pens, where clean water and mineral-salt blocks were available at all times. The diet was offered to the animals twice dairy in the morning (07:00a.m.) and afternoon (04.00p.m.). The liveweight of each cattle was weighed at the beginning and the end of each period to calculate feed intake. Feed provided and refusals were measured daily throughout the experimental period. Feed samples were collected twice a week for DM analysis. Samples of feeds including concentrate, FMS, rice straw, feces were collected randomly daily during the last 7 days of each period. A daily sample of feces of each animal (about 100 g) was collected to be analyses [38]. Urinary samples were collected and prepared for storage and later analyzed for total nitrogen [38], and total purine derivatives and calculation of microbial N supply (MNS) [43, 44]. Details of sampling procedures of rumen fluid from each animal analysis of volatile fatty acids (VFA) [42], protozoal population count [45], and estimated methane (CH_4_) production [46] are presented in details in Wanapat et al. [47]. Blood samples (about 10 ml) were collected from the jugular vein at each rumen sampling time and kept in the tubes to which 0.1 g EDTA was added for analysis of blood urea-nitrogen (BUN) [48].

### Data management and statistical analysis

The samples were estimated according to the statistical design used 4 × 4 Latin Square Design.

All data were included in all analysis subjected to ANOVA according to a 4 × 4 Latin square design using the General Linear Models (GLM) procedures [49]. The results were presented as mean values with the standard error of the means. Difference among means with *P <* 0.05 was accepted as statistical differences while 0.05 < *P* < 0.10 was accepted as a tendency. Treatment means were statistically compared by Duncan’s New Multiple Range Test [50].

## Data Availability

All experimental data are responsible and available under the holding of the corresponding author.

## Abbreviations

ADF: Acid detergent fiber
BUN: Blood urea nitrogen
BW: Body weight
C: Concentrate
CP: Crude protein
C_2_: Acetate
C_3_: Propionate
C_4_: Butyrate
CP: Condensed tannins
DM: Dry matter
EMNS: Efficiency of microbial nitrogen supply
FMS: *Flemingia macrophylla* silage
NDF: Neutral detergent fiber
NH_3_-N: Ammonia-nitrogen
N: Nitrogen
MNS: Microbial nitrogen supply;
OM: Organic matter
PD: Purine derivatives
SP: Saponins
VFA: Volatile fatty acid
